# Boosting CO_2_ Reduction with Spinel CoAl_2_O_4_ Anchored on N-Doped Graphitic Carbon

**DOI:** 10.3390/nano16070422

**Published:** 2026-03-31

**Authors:** Fei Lv, Jitao Shang, Yali Mao, Jianfeng Liu, Xue Bai, Shasha Wei, Yayun Zheng, Teng Wang, Yan Zhao

**Affiliations:** 1 School of Integrated Circuits, Wuhan University, Wuhan 430072, China; feilvv@whu.edu.cn (F.L.); 2021206520037@whu.edu.cn (Y.M.); 2023106520026@whu.edu.cn (J.L.); xuebai@whu.edu.cn (X.B.); 2 Department of Chemical & Materials Engineering, Faculty of Engineering, The University of Auckland, Auckland 1010, New Zealand; jsha302@aucklanduni.ac.nz; 3 School of Science and Engineering, The Chinese University of Hong Kong, Shenzhen 518172, China; weishasha@cuhk.edu.cn; 4 The Institute of Laser Manufacturing, Henan Academy of Sciences, Zhengzhou 450046, China; zhengyayun@hnas.ac.cn; 5 School of Materials Science and Engineering, Wuhan University of Technology, Wuhan 430070, China; t.wang@whu.edu.cn; 6 College of Materials Science and Engineering, Sichuan University, Chengdu 610065, China

**Keywords:** photocatalytic CO_2_ reduction, CoAl_2_O_4_, nitrogen-doped carbon, spinel structure, charge separation

## Abstract

Efficient charge transfer and effective separation of photo-generated charge carriers are pivotal to the photocatalytic process. In this study, a novel CoAl_2_O_4_@nitrogen-doped graphitic carbon (CoAl_2_O_4_@NGC) composite photocatalyst was fabricated via a stepwise hydrothermal method coupled with high-temperature calcination, and its photocatalytic performance for CO_2_ reduction was systematically investigated. X-ray diffraction (XRD), transmission electron microscopy (TEM), X-ray photoelectron spectroscopy (XPS) and photoelectrochemical measurements were employed to characterize the phase structure, microstructure, surface chemical state and photoelectrochemical properties of the catalyst. Spinel-structured CoAl_2_O_4_ nanoparticles were uniformly anchored on the NGC substrate, forming a well-integrated composite interface. XPS analysis confirmed the coexistence of Co^2+^/Co^3+^ mixed valence states in CoAl_2_O_4_ which provides abundant redox sites for CO_2_ activation. Photocatalytic tests showed that CoAl_2_O_4_@NGC exhibits excellent catalytic activity and cycling stability, with CO and CH_4_ yields of 27.88 μmol·g^−1^·h^−1^ and 23.90 μmol·g^−1^·h^−1^, respectively. The narrow bandgap (1.54 eV) enhances visible light absorption, while efficient electron-hole separation and reduced charge transfer resistance improve photocatalytic efficiency. Theoretical calculations further reveal that CoAl_2_O_4_@NGC lowers the adsorption free energy of CO_2_ and the energy barrier for COOH formation, thus facilitating the photocatalytic CO_2_ reduction. This work provides insights for the design of efficient and stable photocatalysts for CO_2_ reduction and deepens the understanding of the synergistic catalytic mechanism in the spinel/nitrogen-doped carbon composite system.

## 1. Introduction

Excessive consumption of fossil fuels has not only exacerbated the energy crisis but also led to a continuous rise in atmospheric carbon dioxide (CO_2_) levels, triggering a series of global environmental and climate issues [1,2,3,4,5]. In this context, converting CO_2_ into high-value fuels or chemicals is considered one of the effective ways to alleviate energy pressure and achieve the goal of carbon neutrality [6,7,8]. Among various CO_2_ conversion strategies, photocatalytic CO_2_ reduction technology has attracted considerable research attention due to its unique advantages of directly utilizing solar energy to drive reactions under mild conditions, which simulates the natural photosynthesis process and serving as an artificial photosynthetic paradigm [9,10,11,12].

Spinel-type transition metal oxides (such as CoAl_2_O_4_) have shown considerable potential in the photocatalysis field due to their unique crystal structure, excellent chemical stability, and favorable visible light response [13,14,15,16]. The coexistence of Co^2+^ and Co^3+^ mixed valence states in their lattice forms abundant redox active sites, which facilitate the adsorption and activation of CO_2_ molecules [17,18]. However, pure spinel materials are often limited by rapid recombination of photo-generated charge carriers and sluggish surface reaction kinetics, resulting in low photocatalytic quantum efficiency that cannot meet the requirements of practical applications [19,20].

To address these challenges, combining spinel with highly conductive carbon-based materials has proven to be an effective modification strategy [21,22,23,24,25]. In particular, nitrogen-doped graphitic carbon (NGC) not only inherits the advantages of carbon materials such as high surface area and porous structure, but the introduction of nitrogen atoms can also effectively modulate the electronic structure of the carbon backbone, enhancing the affinity for reactant molecules and improving electronic conductivity [26,27]. Therefore, the combination of CoAl_2_O_4_ and NGC is expected to realize the enhancement of light absorption, the promotion of efficient charge carrier separation, and facilitating surface reaction kinetics through synergistic effects between the two components, thus improving the photocatalytic CO_2_ reduction performance of the composite material.

Based on these considerations, a CoAl_2_O_4_@NGC composite photocatalyst was designed and constructed in this study via a stepwise hydrothermal-calcination method: first, a 3D honeycomb-like NGC substrate was synthesized; then, CoAl-layered double hydroxide (CoAl-LDH) precursors were grown in situ on the NGC surface; finally, the CoAl-LDH@NGC was calcined to convert CoAl-LDH into spinel-structured CoAl_2_O_4_, thus obtaining the target CoAl_2_O_4_@NGC composite. The phase structure, microstructure, surface chemical state, and photoelectrochemical properties of the catalyst were systematically characterized by various analytical techniques. Meanwhile, the photocatalytic performance and mechanism of CoAl_2_O_4_@NGC for CO_2_ reduction were explored by combining experimental results with density functional theory (DFT) theoretical calculations. This study aims to reveal the structure-activity relationship in the spinel/nitrogen-doped carbon composite system, and provide theoretical and experimental references for the development of efficient photocatalysts for CO_2_ energy conversion.

## 2. Materials and Methods

Detailed characterization procedures and photocatalytic experimental setup are provided in the Supplementary Materials.

### 2.1. Synthesis of 3D Honeycomb-like NGC

Cadmium nitrate tetrahydrate (Cd(NO_3_)_2_·4H_2_O) and polyvinylpyrro-lidone (PVP) were dissolved in deionized water, and the mixture was stirred at room temperature for 12 h to ensure thorough mixing. The obtained homogeneous solution was dried at 95 °C until it was completely converted into a yellow solid block, which was denoted as the NGC precursor. The precursor was transferred to a tubular furnace, heated to 700 °C at a rate of 5 °C/min under a nitrogen atmosphere, and maintained at this temperature for 1 h for carbonization. After the carbonization, the sample was naturally cooled to room temperature in the furnace to obtain the 3D honeycomb-like NGC precursor.

### 2.2. Synthesis of CoAl-LDH@NG

Cobalt nitrate hexahydrate (Co(NO_3_)_2_·6H_2_O, 0.8 mmol), aluminum nitrate nonahydrate (Al(NO_3_)_3_·9H_2_O, 0.4 mmol), of hexamethylenetetramine (250 mg), and the as-prepared NGC precursor (30 mg) were dissolved in 30 mL of methanol, and the mixture was stirred for 1 h to achieve complete dissolution. The resulting solution was transferred to a 100 mL stainless steel autoclave lined with polytetrafluoroethylene (PTFE). The autoclave was sealed and heated in a drying oven at 90 °C for 8 h for hydrothermal reaction. After the reaction, the system was naturally cooled to room temperature. The obtained product was centrifuged and thoroughly washed with ethanol to remove residual inorganic salts and impurities, and then dried in air at 60 °C for 12 h to obtain the CoAl-LDH@NGC composite.

### 2.3. Synthesis of CoAl_2_O_4_@NGC

The as-prepared CoAl-LDH@NGC precursor powder was placed into a crucible and transferred to a muffle furnace. The precursor was heated to 550 °C at a rate of 5 °C/min in air and maintained at this temperature for 1 h for calcination. After natural cooling to room temperature, the final CoAl_2_O_4_@NGC catalyst was obtained.

### 2.4. Density Functional Theory Calculations

All calculations were performed using the CASTEP module in Materials Studio 2020 with the Perdew-Burke-Ernzerhof (PBE) functional adopted to describe the exchange-correlation energy. The key calculation parameters were set as follows:

- Energy cutoff: 500 eV
- Monkhorst-Pack k-point grid: 1 × 1 × 1
- Convergence criteria: Energy tolerance = 1.0 × 10^−5^ eV/atom; force tolerance = 0.03 eV/Å
- Vacuum layer: a 20 Å vacuum layer was added in the z-direction to avoid the periodic boundary interaction between adjacent supercells

## 3. Results and Discussion

### 3.1. Morphological and Structural Characteristics

In this study, we selected tetrahydrate cadmium nitrate (Cd(NO_3_)_2_·4H_2_O) as a metal-based precursor, with the main purpose of utilizing the templating effect of metal salts to assist in the construction of a 3D honeycomb-like nitrogen-doped graphitic carbon (NGC) structure. Specifically, after cadmium nitrate dissolves with polyvinylpyrrolidone (PVP) in deionized water, a stable composite is formed through hydrothermal reaction. During the high-temperature calcination process, cadmium nitrate decomposes into cadmium oxide (CdO) or volatile gases, and does not remain in the final catalyst. Therefore, cadmium primarily serves as a templating material, helping to form the porous carbon structure under high-temperature conditions, rather than as a metal component in the final catalyst. Similar metal salt templating strategies have been validated in several studies. For instance, Gan et al. proposed using metal salts (such as magnesium salts) as templates to effectively construct porous nitrogen-doped carbon materials, achieving control over the pore structure and catalytic performance of the carbon materials via the templating effect [28]. Additionally, He et al. reported the use of metal salts as templates to assist in the synthesis of nitrogen-doped carbon materials with a high surface area [29].

To verify whether cadmium remains in the final material, we performed XPS analysis on the samples. The full XPS spectrum (Figure S1a) shows distinct peaks for carbon (C), nitrogen (N), and oxygen (O) elements, but no signal for cadmium (Cd), indicating that cadmium was completely removed during the calcination process. The C 1s spectrum (Figure S1b) further reveals the presence of C–C, C=N, and C–N structures in the graphitic carbon framework, while the N 1s spectrum (Figure S1c) shows the presence of graphitic-N and pyridinic-N nitrogen species, confirming the effectiveness of nitrogen doping. Moreover, the oxygen signal primarily comes from surface-adsorbed water, which is consistent with the expected properties of nitrogen-doped carbon materials.

**Figure 1:**
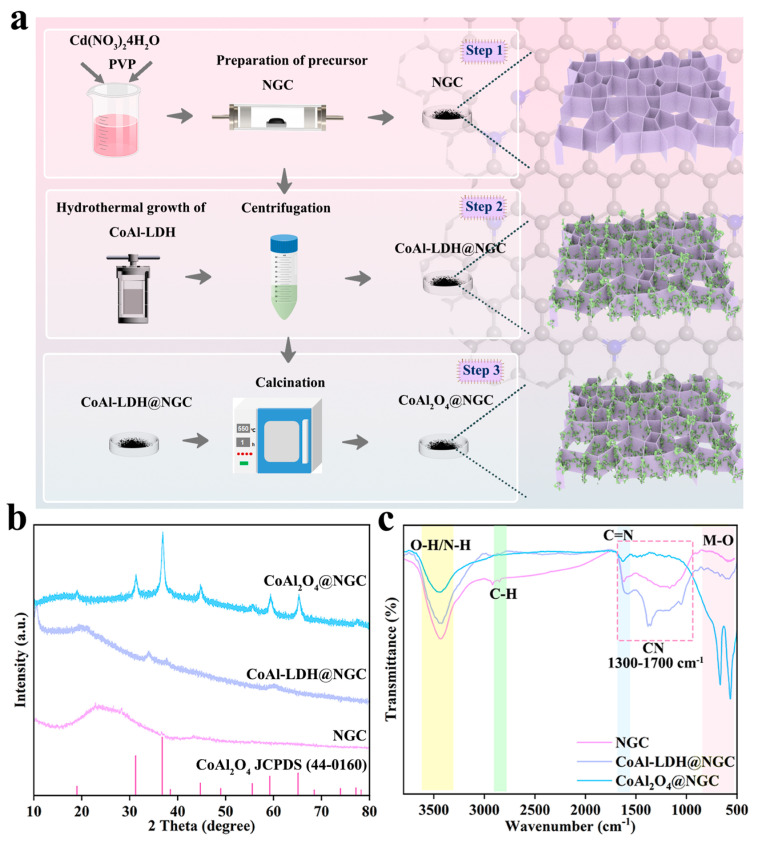
(**a**) Schematic synthetic route of the CoAl_2_O_4_@NGC photocatalyst; (**b**) the XRD patterns and (**c**) the FTIR spectra of NGC, CoAl-LDH@NGC and CoAl_2_O_4_@NGC.

Figure 1a shows the synthetic route of the CoAl_2_O_4_@NGC photocatalyst. Firstly, 3D honeycomb-like NGC was synthesized by calcination of the PVP/Cd(NO_3_)_2_·4H_2_O precursor under a nitrogen atmosphere; then, the CoAl-LDH@NGC nanosheets were grown in situ on the NGC surface via a hydrothermal method; finally CoAl-LDH was converted into the spinel structure CoAl_2_O_4_ through high-temperature calcination, thus obtaining the CoAl_2_O_4_@NGC composite with a well-integrated interface.

The crystal structures of NGC, CoAl-LDH@NGC and CoAl_2_O_4_@NGC were characterized using X-ray diffraction (XRD), and the results are shown in Figure 1b. For pure NGC, no obvious diffraction peaks are observed, indicating its amorphous or low-crystallinity characteristic. CoAl-LDH@NGC exhibits the characteristic diffraction peaks of CoAl-LDH, confirming the successful loading of CoAl-LDH on the NGC surface to form a composite structure. After calcination, the diffraction peaks of CoAl_2_O_4_@NGC are well consistent with the standard card of spinel CoAl_2_O_4_ (JCPDS 44-0160), and the characteristic peaks become became sharp, which verifies the conversion of CoAl-LDH into highly crystalline CoAl_2_O_4_ spinel.

Fourier-transform infrared (FTIR) spectroscopy was used to analyze the surface functional groups and chemical bonding states of the samples, and the results are presented in Figure 1c. For pure NGC, the absorption peaks at ~2920 cm^−1^ and ~2850 cm^−1^ are attributed to the stretching vibration of C-H bonds, the peak at ~1630 cm^−1^ corresponds to the C=N stretching vibration, and the broad peak at ~3400 cm^−1^ is assigned to the O-H/N-H stretching vibration of surface hydroxyl groups and adsorbed water, confirming the successful preparation of nitrogen-doped graphitic carbon with rich surface functional groups [30]. For CoAl-LDH@NGC, the intensity of the O-H/N-H absorption peaks was reduced as compared to NGC, but much stronger than that of CoAl_2_O_4_@NGC [31]. For CoAl_2_O_4_@NGC, the C=N characteristic peak at ~1630 cm^−1^ is still retained, indicating that the NGC skeleton is not destroyed during calcination; meanwhile, new absorption peaks appear in the low-frequency region (~500–700 cm^−1^), which are assigned to the stretching vibration of M-O (Co-O/Al-O) bonds in spinel CoAl_2_O_4_, confirming the successful formation of metal oxide active phases and the conversion of CoAl-LDH to CoAl_2_O_4_ during calcination.

**Figure 2:**
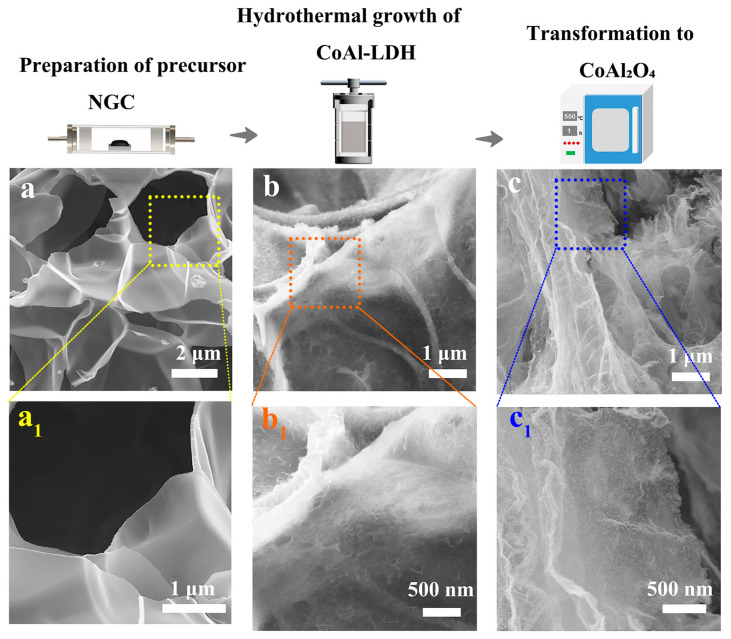
SEM images of (**a**) NGC, (**b**) CoAl-LDH@NGC and (**c**) CoAl_2_O_4_@NGC; (**a_1_**–**c_1_**) Magnified images of the boxed regions in (**a**–**c**), respectively.

Figure 2 shows the scanning electron microscopy (SEM) images of NGC, CoAl-LDH@NGC, and CoAl_2_O_4_@NGC. As can be seen from Figure 2a,a_1_ NGC exhibits a 3D honeycomb-like porous structure with pores of varying sizes uniformly distributed across the matrix. This porous structure is formed by the release of gaseous products during the precursor carbonization process, which can provide a large specific surface area for the loading of active components, and facilitate electron conduction and the diffusion of reactant molecules. For CoAl-LDH@NGC (Figure 2b,b_1_), CoAl-LDH nanosheets are uniformly deposited on the surface and pore walls of NGC, forming a tight layered composite structure. The close combination between CoAl-LDH and NGC constructs a well-integrated interface. After calcination, the surface morphology of CoAl_2_O_4_@NGC underwent significant changes (Figure 2c,c_1_): the matrix shows a slightly curled shape, and the surface became more uniform and dense. The CoAl-LDH nanosheets are converted into CoAl_2_O_4_ nanoparticles after calcination, which are uniformly dispersed on the NGC substrate without obvious agglomeration. This structural transformation not only improves the crystallinity of CoAl_2_O_4_ but also maintains the porous structure of the composite, which is beneficial to improving the stability and photocatalytic performance of the catalyst.

**Figure 3:**
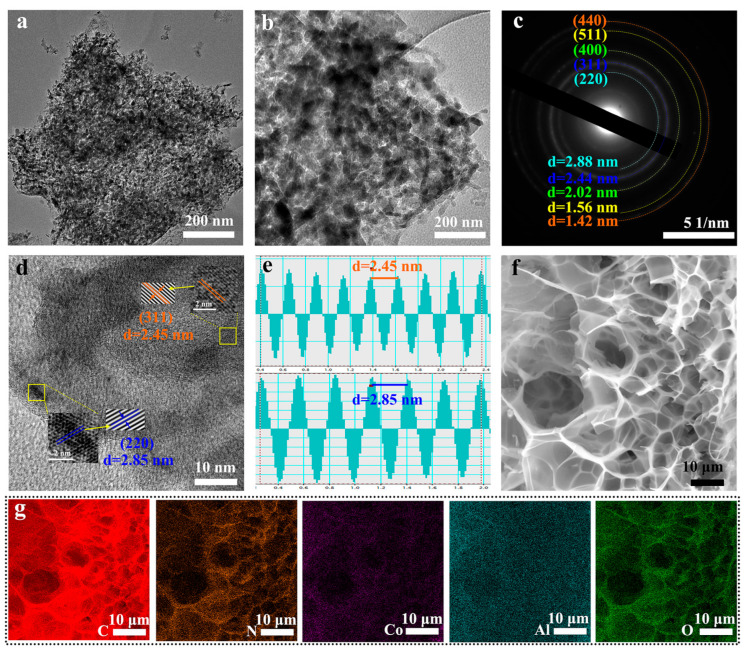
(**a**,**b**) TEM images of CoAl_2_O_4_@NGC; (**c**) SAED; (**d**) HRTEM image; (**e**) Lattice fringe analysis; (**f**,**g**) EDS Mapping images.

Transmission electron microscopy (TEM) and related techniques were used to further characterize the microscopic structure of CoAl_2_O_4_@NGC, and the results are shown in Figure 3. The TEM images in Figure 3a,b show that CoAl_2_O_4_ nanoparticles are uniformly dispersed on the NGC nanosheet substrate, and the composite maintains a favorable porous structure, which provides a stable structural foundation for the photocatalytic reaction. The selected area electron diffraction (SAED) pattern in Figure 3c shows clear diffraction rings corresponding to the crystal planes (220), (311), (400), (440), and (511), confirming that CoAl_2_O_4_@NGC possesses good crystallinity and a typical spinel structure.

High-resolution TEM (HRTEM) images (Figure 3d,e) reveal clear lattice fringes of CoAl_2_O_4_, with the interplanar spacings of 2.85 Å and 2.45 Å corresponding to the (220) and (311) crystal planes of spinel CoAl_2_O_4_, respectively, providing direct structural evidence for the formation of spinel CoAl_2_O_4_. The energy-dispersive X-ray spectroscopy (EDS) elemental mapping images (Figure 3f,g) show that C, N, Co, Al and O elements are uniformly distributed in the CoAl_2_O_4_@NGC composite: N element is uniformly dispersed in the carbon matrix, confirming the successful nitrogen doping of NGC; Co and Al elements are co-located with O element, which verifies the uniform distribution of CoAl_2_O_4_ on the NGC substrate and the successful construction of the CoAl_2_O_4_@NGC composite structure.

**Figure 4:**
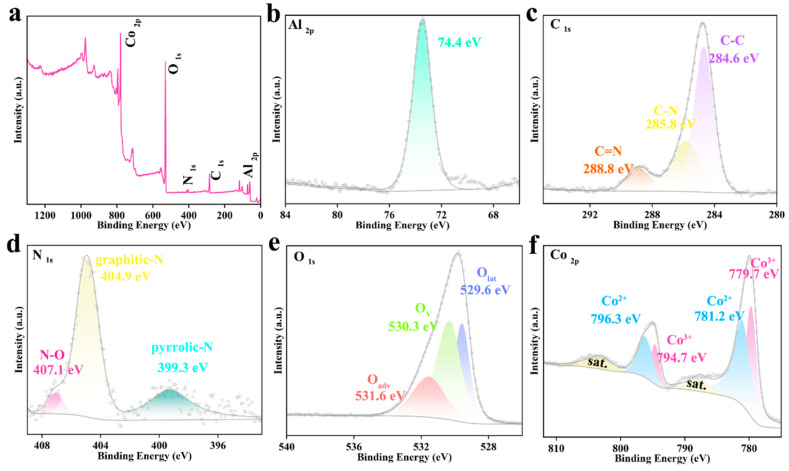
(**a**) Full XPS survey of CoAl_2_O_4_@NGC; XPS spectrum of CoAl_2_O_4_@NGC (**b**) Al 2p, (**c**) C 1s, (**d**) N 1s, (**e**) O 1s, and (**f**) Co 2p.

X-ray photoelectron spectroscopy (XPS) was employed to analyze the surface elemental composition and chemical state of CoAl_2_O_4_@NGC, and the results are shown in Figure 4. The full XPS spectrum scan in Figure 4a clearly detects characteristic photoelectron peaks of Co, Al, O, N, and C, confirming the coexistence of these elements in the catalyst. Peak fitting of the high-resolution spectra for each element further reveals their detailed chemical states: In the Al 2p spectrum (Figure 4b), the characteristic peak at 74.72 eV corresponds to Al^3+^, indicating that aluminum exists in its oxidized form within the spinel structure [32,33,34]. The C 1s spectrum (Figure 4c) shows a complex chemical environment, containing peaks for C–C bonds of sp^2^ hybridized carbon (284.6 eV), as well as C–N (285.8 eV) and C=N (288.8 eV) bonding peaks, providing direct evidence of nitrogen doping into the carbon substrate [35]. Correspondingly, the N 1s spectrum (Figure 4d) further confirms the nitrogen doping form, with peak fitting results showing pyrrolic nitrogen (399.3 eV) and graphitic nitrogen (404.9 eV) [36,37,38]. These nitrogen species play an important role in improving the electronic donor characteristics and conductivity of the carbon material. The O 1s spectrum (Figure 4e) presents an asymmetric peak, which can be fitted into three components at 529.6 eV, 530.3 eV, and 531.6 eV, corresponding to oxygen vacancies (O_vac_), lattice oxygen (O_lat_), and surface adsorbed oxygen or hydroxyl oxygen (O^2−^/OH^−^), respectively [31,39,40]. These abundant oxygen species coordinate with metal sites, forming the active surface of the catalyst. The high-resolution Co 2p spectrum (Figure 4f) shows the typical spin–orbit split double peaks, which, upon fitting, reveal that cobalt exists in both Co^2+^ (approximately 781.2 eV and 796.3 eV) and Co^3+^ (779. 7 eV and 794.7 eV) oxidation states, along with clear satellite peaks [41,42,43,44]. The coexistence of these mixed valence states in CoAl_2_O_4_ is characteristic of the spinel structure and is a key source of its redox activity, potentially serving as effective charge transfer media and active sites in the photocatalytic reaction. The XPS analysis systematically reveals the chemical states of the components in CoAl_2_O_4_@NGC. The NGC substrate provides a conductive network rich in nitrogen functional groups, which helps to enhance electron transfer and reactant adsorption. The multiple valence states of cobalt and the stable oxidation state of aluminum in the spinel phase, combined with the diverse surface oxygen species, create a highly active catalytic interface.

Figure S2 presents a systematic characterization of the pore structure and specific surface area of NGC, CoAl-LDH@NGC, and CoAl_2_O_4_@NGC using N_2_ adsorption–desorption isotherms. As shown in Figure S2a, the isotherm of NGC exhibits a typical Type IV curve with a prominent H3-type hysteresis loop, indicating the presence of a slit-like mesoporous structure mainly formed by the stacking of flaky particles. The pore size distribution (inset) shows that the pore size is mainly concentrated around 1.74 nm, and the calculated specific surface area is 137.34 m^2^/g. This relatively high specific surface area and abundant mesoporous structure are highly beneficial for the adsorption and diffusion of gas molecules, providing a solid structural foundation for the subsequent loading of active components and improving catalytic efficiency. When CoAl-LDH was loaded onto the NGC surface through a hydrothermal method (Figure S2b), the resulting CoAl-LDH@NGC composite material retained the Type IV isotherm with a clear H3-type hysteresis loop, indicating that the primary pore structure was preserved. However, its specific surface area slightly decreased to 126.03 m^2^/g, and the pore size slightly decreased to around 1.70 nm. This is primarily due to the growth of CoAl-LDH nanosheets, which occupied some of the pore spaces or covered some of the pore entrances. Despite this, the composite material still maintained good pore permeability. After subsequent calcination, the final product CoAl_2_O_4_@NGC (Figure S2c) still exhibited a Type H3 hysteresis loop in the isotherm, confirming that the mesoporous structure was well-maintained despite the high-temperature phase transformation. Notably, its specific surface area further decreased to 107.45 m^2^/g, while the average pore size slightly increased to 1.86 nm. This change can be attributed to the dehydroxylation of the CoAl-LDH sheets during calcination and their transformation into the spinel phase CoAl_2_O_4_, which led to the densification of the original LDH sheets and the formation of new pores at the interfaces, resulting in a decrease in the specific surface area and a slight increase in the average pore size. Overall, although the specific surface area gradually decreased from NGC to CoAl_2_O_4_@NGC, all samples maintained a typical mesoporous structure and considerable specific surface area. This moderately optimized pore structure not only provides ample active site exposure but also facilitates the transport of reactant molecules and the desorption of products. Combined with the high conductivity of the NGC substrate and the catalytic activity of CoAl_2_O_4_, this structural design creates a favorable microenvironment for the photocatalytic CO_2_ reduction reaction.

### 3.2. Photocatalytic CO_2_ Reduction Performance

**Figure 5:**
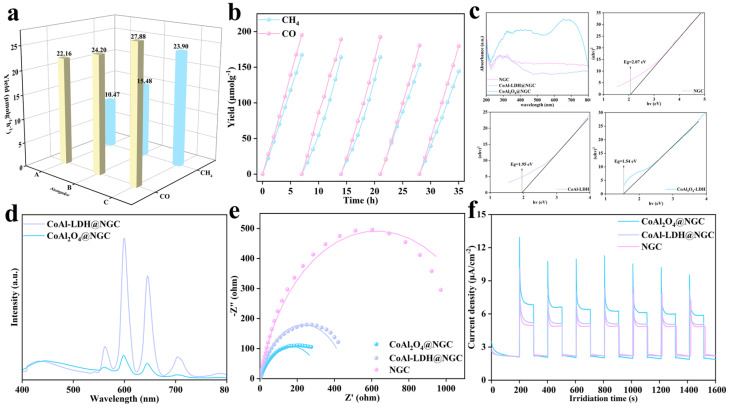
(**a**) Photocatalytic CO_2_ reduction activities over A: NGC, B: CoAl-LDH@NGC and C: CoAl_2_O_4_@NGC; (**b**) The recyclability of CoAl_2_O_4_@NGC for the photocatalytic CO_2_ reduction; (**c**) UV-Vis diffuse reflectance spectra and plots of (αhv)^2^ versus energy (hv) of all photocatalysts; (**d**) The Steady-state PL spectrum of CoAl-LDH@NGC and CoAl_2_O_4_@NGC; (**e**) Electrochemical impedance spectra and (**f**) Transient photocurrent responses of the all photocatalysts.

A series of photoelectrochemical characterizations were conducted to evaluate the photocatalytic CO_2_ reduction performance and intrinsic mechanism of the prepared catalysts, and the results are shown in Figure 5. Figure 5a shows the product yield of different catalysts in the photocatalytic CO_2_ reduction reaction. The unloaded NGC substrate can generate substantial amounts of CO and CH_4_ in photocatalytic CO_2_ reduction. Despite the absence of an external charge separation agent, the nitrogen doping sites introduced into NGC can still promote the effective separation of photogenerated electrons and holes, thereby driving the CO_2_ reduction reaction. This is primarily attributed to the fact that the introduction of nitrogen atoms not only enhances the visible light absorption of the carbon material but also provides effective trap sites for photogenerated carriers. Therefore, even though the charge separation efficiency of NGC itself is relatively limited, significant generation of CO and CH_4_ can still be achieved. The results clearly show that CoAl_2_O_4_@NGC exhibits the best catalytic activity, with a CO yield of 27.88 μmol·g^−1^·h^−1^ and a CH_4_ yield of 23.90 μmol·g^−1^·h^−1^, significantly higher than those of NGC and CoAl-LDH@NGC. This indicates that after high-temperature calcination, CoAl-LDH was successfully transformed into CoAl_2_O_4_ with a spinel structure, and this structural transformation likely optimized the electronic structure of the catalyst and surface active sites, significantly enhancing the photocatalytic CO_2_ reduction efficiency.

Figure 5b shows the stability test results of CoAl_2_O_4_@NGC in multiple photocatalytic CO_2_ reduction reaction cycles. The experiments demonstrate that, after several reaction cycles, the product yield of CoAl_2_O_4_@NGC did not decrease significantly and maintained high catalytic activity, indicating excellent reaction stability and structural durability. This good cycling performance suggests that CoAl_2_O_4_@NGC has a strong ability to resist photo-corrosion and structural degradation during long-term operation, making it a promising photocatalyst for practical applications.

Light absorption capability is one of the key factors determining the performance of semiconductor photocatalysts. Figure 5c shows the UV-Vis diffuse reflectance spectra of all catalysts, and the Tauc plot in the inset was used to calculate the bandgap values of each sample. From the spectral data, it can be seen that CoAl_2_O_4_@NGC exhibits a wider absorption range from ultraviolet to visible light, with significantly enhanced absorption intensity in the visible light region. The bandgap values, determined from the Tauc plot, are 2.07 eV for NGC, 1.95 eV for CoAl-LDH@NGC, and 1.54 eV for CoAl_2_O_4_@NGC. The significant narrowing of the bandgap means that CoAl_2_O_4_@NGC can more effectively utilize the visible light component of the solar spectrum, generating more photo-induced electron-hole pairs. This is a crucial factor contributing to its enhanced photocatalytic performance.

The separation efficiency of photo-induced charges directly affects the photocatalytic quantum efficiency. Figure 5d compares the steady-state photoluminescence (PL) spectra of CoAl-LDH@NGC and CoAl_2_O_4_@NGC. PL intensity is an important indicator that reflects the recombination process of photogenerated electrons and holes. Specifically, lower PL intensity typically indicates less recombination of photogenerated electrons and holes, meaning that the efficiency of electron and hole separation on the catalyst surface is higher. In contrast, higher PL intensity usually suggests that electrons and holes are more likely to recombine, leading to photogenerated charge carriers not effectively participating in the reaction, thereby reducing the photocatalytic efficiency. The results show that the PL emission intensity of CoAl_2_O_4_@NGC is significantly lower than that of CoAl-LDH@NGC. A lower PL intensity typically indicates a lower recombination rate of photo-induced electrons and holes, which corresponds to higher separation efficiency. Therefore, this result suggests that CoAl_2_O_4_@NGC achieves more effective charge separation, allowing more photo-generated carriers to participate in surface redox reactions, thus improving the efficiency of photocatalytic CO_2_ reduction.

Interface charge transfer dynamics is another key factor affecting the photocatalytic reaction rate. Figure 5e shows the electrochemical impedance spectra (EIS) of all catalysts. The radius of the Nyquist plot arc reflects the charge transfer resistance at the electrode/electrolyte interface. It can be observed that CoAl_2_O_4_@NGC exhibits the smallest arc radius, indicating the lowest charge transfer resistance and optimal electronic conductivity. This means that during the photocatalytic reaction, CoAl_2_O_4_@NGC can transfer photo-induced electrons from the bulk to the surface more quickly, effectively suppressing bulk recombination and thereby enhancing the catalytic reaction kinetics.

Transient photocurrent response is a direct method to evaluate the separation and migration capability of photo-generated carriers. Figure 5f shows the photocurrent response curves of all catalysts under intermittent light illumination. CoAl_2_O_4_@NGC exhibits the most significant photocurrent density, much higher than NGC and CoAl-LDH@NGC. This result further confirms that CoAl_2_O_4_@NGC has higher photo-generated carrier separation efficiency and faster interface charge migration rates. The stronger photocurrent response is not only related to the enhanced light absorption due to its narrow bandgap but also to its optimized electronic structure and good conductivity. Upon illumination, the photocurrent curve initially displays a sharp peak, which gradually decays toward a steady-state value. This behavior is attributed to the rapid generation and separation of photogenerated electrons and holes. NGC, with its exceptional conductivity and electronic modulation from pyridinic and graphitic nitrogen, facilitates efficient charge carrier separation and transport, resulting in a rapid surge in current. When combined with a co-catalyst, the difference in Fermi levels at the interface creates a space charge region, further amplifying the initial photocurrent. The subsequent rapid decay reflects a kinetic competition: the trap states introduced by nitrogen doping are quickly filled, causing a decrease in free charge carrier concentration, while any mismatch between the catalytic reaction rate and charge extraction rate leads to charge accumulation at the interface, promoting electron-hole recombination and ultimately driving the current back to the steady-state value.

Based on the Mott-Schottky electrochemical analysis, the NGC and CoAl_2_O_4_ samples exhibit distinct semiconductor properties and electronic structural characteristics. As shown in Figure S3a, the Mott-Schottky curve of the NGC sample has a positive slope, with the flat-band potential around −1.01 V (vs. Ag/AgCl), corresponding to a Fermi level of approximately −3.63 eV when converted to the vacuum energy level. In contrast, the CoAl_2_O_4_ sample shows a negative slope in its Mott-Schottky curve, with the flat-band potential around 0.90 V (vs. Ag/AgCl), corresponding to a Fermi level of approximately −5.54 eV (Figure S3b). The significant difference in the flat-band potentials reveals an essential distinction in the Fermi level positions, with NGC having a higher Fermi level and CoAl_2_O_4_ a lower one. Upon contact between the two materials, electrons spontaneously migrate from the higher Fermi level of NGC to the lower Fermi level of CoAl_2_O_4_, forming a built-in electric field from NGC to CoAl_2_O_4_ at the interface. This built-in electric field effectively drives the spatial separation of photogenerated charge carriers, with photogenerated electrons accumulating on the CoAl_2_O_4_ side and photogenerated holes on the NGC side, thereby achieving spatial separation of charge carriers in the catalyst.

**Figure 6:**
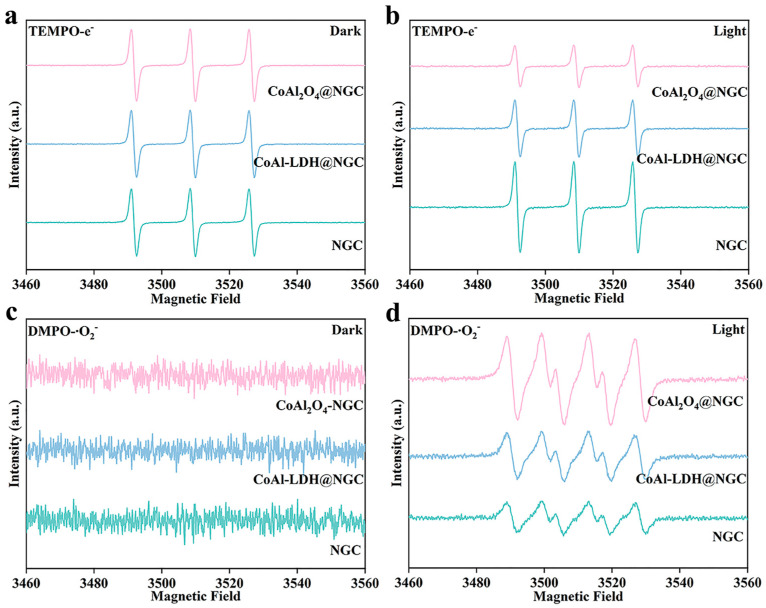
EPR spectra of the catalysts: (**a**) TEMPO–e^−^ signal detected before illumination; (**b**) TEMPO–e^−^ signal detected after illumination; (**c**) DMPO–·O_2_^−^ signal detected before illumination; (**d**) DMPO–·O_2_^−^ signal detected after illumination.

Through electron paramagnetic resonance (EPR) spectroscopy, the key active species generated during the photocatalytic process in different catalysts were systematically studied. Figure 6a shows the EPR signal of the TEMPO–e^−^ adduct captured under dark conditions. No significant signals were detected under dark conditions in all catalyst systems, indicating that no photogenerated electrons were produced in the absence of light. This phenomenon further confirms the strong dependence of photogenerated electron generation and separation on illumination conditions. Under illumination (Figure 6b), the CoAl_2_O_4_@NGC catalyst exhibited the strongest TEMPO–e^−^ signal, suggesting that this catalyst can effectively promote the generation and separation of photogenerated electrons and transfer them to surface active sites. In comparison, the signal intensities for CoAl-LDH@NGC and NGC were significantly lower, indicating lower efficiency of photogenerated electron generation and separation, with more severe electron-hole recombination, which limits their efficiency in photocatalytic reactions. Figure 6c shows the EPR signal of the DMPO–O_2_^−^ adduct under dark conditions. In the absence of light, no significant superoxide radical (·O_2_^−^) signals were detected in any of the catalyst systems, indicating that the generation of ·O_2_^−^ radicals is weak or absent under dark conditions. This further demonstrates the importance of illumination for the generation of ·O_2_^−^ radicals. Under illumination (Figure 6d), CoAl_2_O_4_@NGC exhibited the strongest DMPO–O_2_^−^ signal, indicating that this catalyst can effectively promote the transfer of photogenerated electrons and facilitate the conversion of oxygen molecules into superoxide radicals (·O_2_^−^). In contrast, CoAl-LDH@NGC and NGC showed significantly weaker signals, reflecting their lower efficiency in the electron transfer process.

In summary, the CoAl_2_O_4_@NGC catalyst demonstrates excellent overall performance in the photocatalytic CO_2_ reduction reaction, and its superior performance can be attributed to the following aspects: (1) The narrowed bandgap (1.54 eV) extends the visible light absorption range, improving light energy utilization efficiency; (2) Efficient charge separation (low PL intensity) ensures that more photo-generated carriers can migrate to the surface and participate in the reaction; (3) Low charge transfer resistance (small EIS arc radius) and strong photocurrent response further confirm its excellent carrier dynamics. These synergistic effects of the photoelectronic properties allow CoAl_2_O_4_@NGC to achieve both high activity and stability in photocatalytic CO_2_ reduction.

### 3.3. Photocatalytic CO_2_ Reduction Mechanism

**Figure 7:**
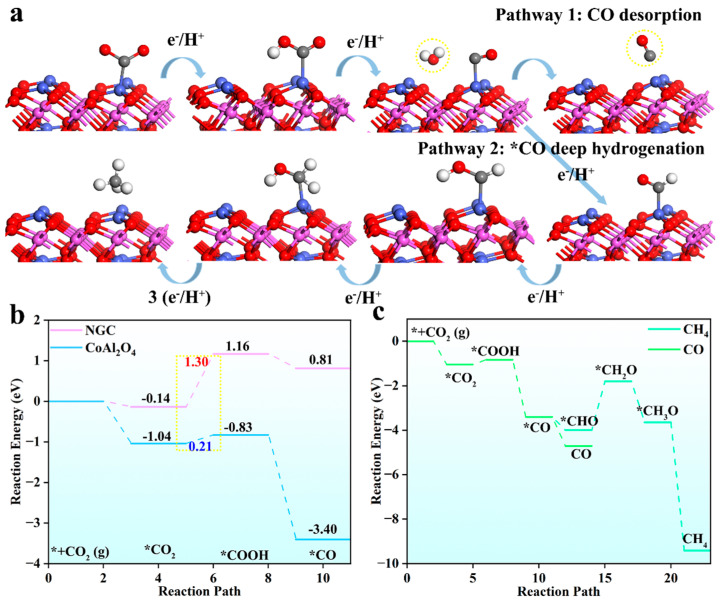
(**a**) Schematic diagram of the photocatalytic CO_2_ reduction reaction pathways on the CoAl_2_O_4_@NGC surface; (**b**) Comparison of the Gibbs free energy (ΔG) between CoAl_2_O_4_@NGC and NGC; (**c**) Gibbs free energy diagrams for the two reaction pathways of CO_2_ photocatalytic reduction to CH_4_ and CO on CoAl_2_O_4_@NGC.

To gain a deeper understanding of the microscopic mechanism of CoAl_2_O_4_@NGC in the photocatalytic CO_2_ reduction reaction, theoretical DFT calculations were employed to explore the possible reaction pathways. Based on the detected intermediate products, we propose a pathway for the conversion of CO_2_ into methane and carbon monoxide, as shown in Figure 7a. This reaction network mainly involves two competing pathways: the first pathway is the CO desorption pathway (Pathway 1), where CO_2_ molecules are reduced to CO intermediates on the catalyst surface via stepwise proton-coupled electron transfer, and CO desorbs directly from the catalyst surface, releasing gaseous CO products. The second pathway is the CO deep hydrogenation pathway (Pathway 2), where the CO intermediate does not desorb but continues to receive protons and electrons, undergoing a deep hydrogenation reaction to ultimately produce CH_4_. The competition between these two pathways directly determines the product selectivity, and the charge transfer and proton-coupling efficiency involved in the reaction are crucial for improving the overall photocatalytic quantum efficiency.

To further elucidate the intrinsic reasons behind the enhanced catalytic performance of spinel CoAl_2_O_4_, we analyzed the Gibbs free energy changes in the key intermediate products, as shown in Figure 7b, comparing the energy differences between the CO_2_ reduction process on the CoAl_2_O_4_@NGC surface and on pure NGC. The results show that the adsorption free energy of CO_2_ on the CoAl_2_O_4_@NGC surface is significantly lower than that on the NGC surface, highlighting the key role of the CoAl_2_O_4_ component in enhancing CO_2_ molecule capture and activation. More importantly, CoAl_2_O_4_@NGC significantly lowers the energy barrier for the key rate-limiting step from CO_2_ to COOH, making it easier to overcome the initial activation energy barrier. This energy advantage means that photo-generated electrons on the CoAl_2_O_4_@NGC surface can more efficiently inject into adsorbed CO_2_ molecules, promoting their conversion. From a thermodynamic perspective, this explains the substantial enhancement in its photocatalytic reduction efficiency.

Figure 7c further focuses on the Gibbs free energy changes for the two competing pathways on the CoAl_2_O_4_@NGC surface. By comparing the full free energy curves for CH_4_ and CO generation, it can be seen that the key step energy penalties for both reaction pathways on CoAl_2_O_4_@NGC remain at relatively low levels. Notably, the energy comparison at the CO intermediate branch point reveals that the Gibbs free energy change for CO desorption to form CO is lower than the energy barrier for further deep hydrogenation. This thermodynamic advantage means that the reaction intermediate is more likely to desorb from the catalyst surface rather than undergo further hydrogenation, thus making CO the dominant product. This theoretical prediction aligns well with the experimental yield results in Figure 5a, where the CO yield (27.88 μmol·g^−1^·h^−1^) is slightly higher than CH_4_ (23.90 μmol·g^−1^·h^−1^), and both contribute as major products.

The excellent performance of CoAl_2_O_4_@NGC in the photocatalytic CO_2_ reduction reaction can be attributed to its synergistic mechanism across the entire process, from light absorption, charge behavior, to surface reaction dynamics: (1) CoAl_2_O_4_@NGC has a narrow bandgap, enabling efficient utilization of visible light to generate photo-induced charge carriers; (2) The recombination of photo-generated electron-hole pairs is effectively suppressed, the charge transfer resistance is low, and the photo-generated electrons are efficiently separated and rapidly migrated to the catalyst surface to participate in reactions; (3) Cobalt in CoAl_2_O_4_ possesses mixed valence states, providing redox active sites for CO_2_ activation, while the NGC substrate improves the electron conductivity of the composite; (4) Theoretical calculations further reveal that this catalyst significantly improve the adsorption of CO_2_ and reduce the energy penalties for key intermediates. At the reaction pathway branching point, the CO desorption pathway is more favorable than the deep hydrogenation pathway, which is in good agreement with the experimental results showing a higher yield of CO than CH_4_; (5) CoAl_2_O_4_@NGC retains a well-developed porous structure, providing a favorable microenvironment for the diffusion of reactant molecules and the desorption of products. It is the synergistic effect between the spinel CoAl_2_O_4_ phase and the NGC substrate that enables this catalyst to achieve high activity, good stability, and tunable selectivity in photocatalytic CO_2_ reduction.

## 4. Conclusions

In this study, the CoAl_2_O_4_@NGC catalyst was designed and synthesized, and its performance and underlying mechanism in photocatalytic CO_2_ reduction were systematically investigated through a combination of experimental and theoretical approaches. The results demonstrate that the CoAl_2_O_4_@NGC catalyst exhibits outstanding catalytic performance in the CO_2_ reduction reaction, which can be attributed to several key factors:

1. Optimized catalyst structure: CoAl_2_O_4_ nanoparticles with a well-defined spinel structure are uniformly anchored on the 3D honeycomb-like NGC substrate, forming a well-integrated composite interface. This structural design effectively reduces the adsorption free energy of CO_2_ molecules, optimizes the active sites of the catalyst, facilitates the capture and activation of CO_2_, and thus enhances the catalytic reaction efficiency.
2. Enhanced photoelectric properties: The narrow band gap (1.54 eV) of CoAl_2_O_4_@NGC enables efficient absorption of visible light, leading to the generation of a large number of photogenerated electron–hole pairs. The lower PL emission intensity and smaller EIS arc radius indicate that the composite has superior charge separation and transfer capabilities, which significantly reduce the recombination of photogenerated carriers and thus improve the photocatalytic efficiency.
3. Abundant active sites: The coexistence of Co^2+^/Co^3+^ mixed valence states in the spinel CoAl_2_O_4_ and the nitrogen doping in the carbon matrix jointly construct a highly active catalytic interface, providing ample redox sites for CO_2_ activation and electron transfer channels for the photocatalytic reaction.
4. Excellent stability and recyclability: The CoAl_2_O_4_@NGC catalyst demonstrates excellent cycling stability in photocatalytic CO_2_ reduction; after multiple reaction cycles, the product yields remain stable, indicating the catalyst’s outstanding durability and long-term operational stability.

This work not only elucidates the synergistic catalytic mechanism of the spinel CoAl_2_O_4_@NGC composite interface in photocatalytic CO_2_ reduction but also provides valuable insights for the rational design of novel, efficient, and stable photocatalysts for CO_2_ energy conversion and environmental remediation applications.

## Data Availability

The original contributions presented in this study are included in the article/Supplementary Materials. Further inquiries can be directed to the corresponding author.

## Supplementary Materials

The following supporting information can be downloaded at: https://www.mdpi.com/article/10.3390/nano16070422/s1.
