# Supplementary material for: Boosting CO2 Reduction with Spinel CoAl2O4 Anchored on N-Doped Graphitic Carbon
Source: Nanomaterials (Basel). 2026 Mar 31;16(7):422. doi: 10.3390/nano16070422 (PMC13075167; doi:10.3390/nano16070422)
Supplement: Supplementary file 1 [file nanomaterials-16-00422-s001.zip › nanomaterials-4212079-supplementary.pdf]

Supporting Information for

Boosting CO_2 Reduction with Spinel $CoAl_2O_4$ Anchored on N-Doped Graphitic Carbon

Fei Lv [1], Jitao Shang [2], Yali Mao [1], Jianfeng Liu [1], Xue Bai [1], Shasha Wei [3], Yayun Zheng [4], Teng Wang [5] and Yan Zhao [1,6,*]

[1] School of Integrated Circuits, Wuhan University, Wuhan 430072, China; feilvv@whu.edu.cn (F.L.); 2021206520037@whu.edu.cn (Y.M.); 2023106520026@whu.edu.cn (J.L.); xuebai@whu.edu.cn (X.B.)
[2] Department of Chemical & Materials Engineering, Faculty of Engineering, The University of Auckland, Auckland 1010, New Zealand; jsha302@aucklanduni.ac.nz
[3] School of Science and Engineering, The Chinese University of Hong Kong, Shenzhen 518172, China; weishasha@cuhk.edu.cn
[4] The Institute of Laser Manufacturing, Henan Academy of Sciences, Zhengzhou 450046, China; zhengyayun@hnas.ac.cn
[5] School of Materials Science and Engineering, Wuhan University of Technology, Wuhan 430070, China; t.wang@whu.edu.cn
[6] College of Materials Science and Engineering, Sichuan University, Chengdu 610065, China
* Correspondence: yan2000@whu.edu.cn

**S1**	Materials and chemicals
**S2**	Materials characterizations
**S3**	Photoelectrochemistry measurement
**S4**	Photocatalytic CO_2 reduction
**Table S1**	Temperature change of photocatalytic CO_2 reduction under visible light irradiation
**Figure S1**	(a) Full XPS survey of NGC; XPS spectrum of NGC (b) C 1s and (d) N 1s.
**Figure S2**	(a) N_2 adsorption-desorption isotherms and pore size distribution (inset) of NGC; (b) N_2 adsorption-desorption isotherms and pore size distribution (inset) of CoAl-LDH@NGC; (c) N_2 adsorption-

	desorption isotherms and pore size distribution (inset) of $CoAl_2O_4$@NGC.
**Figure S3**	(a) Mott-Schottky curve of NGC; (b) Mott-Schottky curve of $CoAl_2O_4$.
**Figure S4**	Model for $CoAl_2O_4$@NGC
**Table S2**	**Table S2** Calculation formula of Gibbs free energy

S1 Materials and chemicals

All of the chemical reagents were analytical grade and used without further purification. Cobalt nitrate hexahydrate ($Co(NO_3)_2·6H_2O$), Aluminum nitrate nonahydrate ($Al(NO_3)_3·9H_2O$), cadmium nitrate tetrahydrate ($Cd(NO_3)_2·4H_2O$), hexamethylenetetramine, polytetrafluoroethylene and polyvinylpyrrolidone (PVP) were purchased from Aladdin Reagent Co., Ltd. and carbon dioxide gas (CO_2 99.99 %), carbon monoxide gas (CO 99.90 %), methane gas (CH_4 99.999 %) were purchased from Wuhan Wu Gang Gas Co. Ltd. Deionized (DI) water was used in all experiments.

S2 Materials characterizations

In order to identify the crystal phase of the samples, the powder X-ray diffraction (XRD) patterns of the sample were obtained using Cu Kα radiation source (λ=1.54056 Å) at a scan rate of 5° min^{-1} on a D/MAX-2500 diffractometer (Riga, Japan). Scanning electron microscope (SEM) images were obtained on the MIRA3 field emission SEM. Transmission electron microscope (TEM) images, high-resolution transmission electron microscope (HRTEM) images and selected electron diffraction (STEM) patterns were converged on the JEM-2100 TEM. X-ray photoelectron spectroscopy (XPS) data was obtained by ESCALAB 250Xi. The solid-state UV diffuse reflectance absorption spectrum in the region of 200 ~800 nm was recorded on a Shimadzu 2450 spectrophotometer using $BaSO_4$ as the reflectance standard. Brunauer- Emmett-Teller (BET) surface areas were measured using a JW-BK100B with N_2 adsorption-desorption after drying under vacuum at 150 ℃ for 5 h. Fourier transform infrared spectra (FT-IR) were recorded on a PerkinElmer Spectrum FT-IR spectrometer. In order to

investigate the photoluminescence (PL) properties of the samples, the PL spectra were measured using a FLS 980 spectrofluorometer (Edinburgh Instruments, UK). The excitation source was a 375 nm laser, and the emission spectra were recorded in the range of 400–800 nm. All measurements were performed at room temperature, and the samples were excited with the 375 nm wavelength to capture the emission characteristics of the material. The data was collected with an integration time of 2 s and a slit width of 2 nm to ensure optimal resolution and signal intensity.

S3 Photoelectrochemistry measurement

The electrochemical measurements are carried out on an electrochemical workstation (CHI760C, Chinstruments, China) utilizing a three-electrode setup. The counter electrode is a Pt foil, the reference electrode is an Ag/AgCl electrode, and the working electrode is an FTO conductive glass (11cm) with the conducting side covered with thin sample film. The electrolyte is 0.5 M Na_2SO_4 solution.

S4 Photocatalytic CO_2 reduction

The photocatalytic CO_2 reduction experiment was conducted in a 200 mL customized Pyrex reactor; the two openings of the reactor were sealed with silicone rubber. A 300W Xenon lamp is placed 10 cm above the reactor, serving as the light source that induced the photocatalytic process. In a typical photocatalysis experiment, 5 mL of DI water was added and swirled by ultrasonic for three minutes to make a suspension out of 10 mg of prepared $CoAl_2O_4$@NGC photocatalyst in a glass petri dish with a diameter of 6 cm. The petri dish was dried in the oven at 60 °C. In order to provide a hydrogen source for photocatalytic reduction of CO_2, 500 μL of DI water was

uniformly covered on the surface of the dried photocatalyst. After purging the system with CO_2 at a rate of 30 mL/min for 20 min, turned on the xenon lamp, and the system was kept at 0.8 MPa pressure (all-glass automatic online trace gas analysis system, Labsolar 6A, Beijing Perfectlight Technology Co., Ltd., China). Gas chromatography and flame ionization detection (GC-9790II) were employed to measure products every 30 minutes. The catalytic temperature was maintained at about 25 ° C under light irradiation with cooling water, and the temperature was measured every hour with an IR608A thermometer **(Table S1)**.

Table S1 Temperature change of photocatalytic CO_2 reduction under visible light irradiation

**Test times (h)**	**0**	**1**	**2**	**3**	**4**	**5**	**6**	**7**
**Temperature (°C)**	25.2	25.1	25.0	25.2	25.0	25.1	25.0	25.3

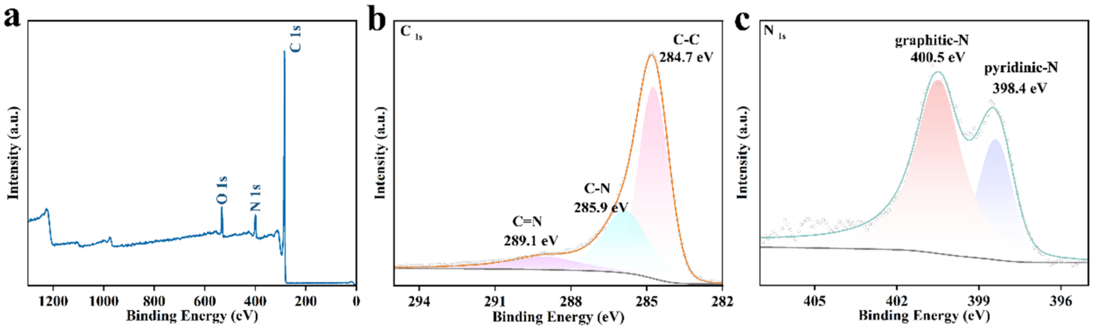

Figure S1. (a) Full XPS survey of NGC; XPS spectrum of NGC (b) C 1s and (c) N 1s.

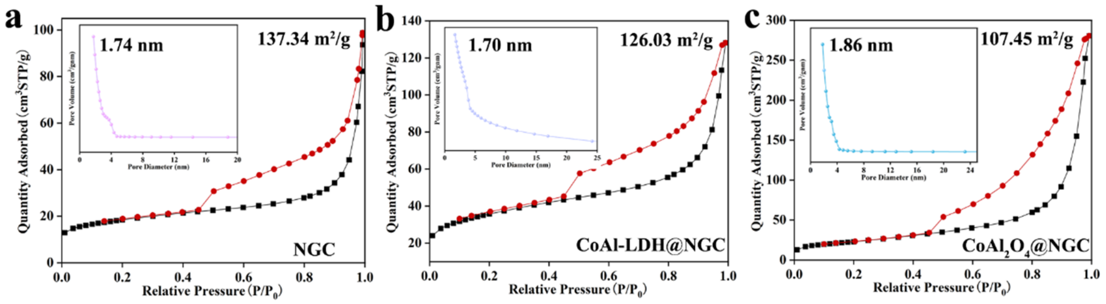

Figure S2. (a) N_2 adsorption-desorption isotherms and pore size distribution (inset) of NGC; (b) N_2 adsorption-desorption isotherms and pore size distribution (inset) of CoAl-LDH@NGC; (c) N_2 adsorption-desorption isotherms and pore size distribution (inset) of $CoAl_2O_4$@NGC.

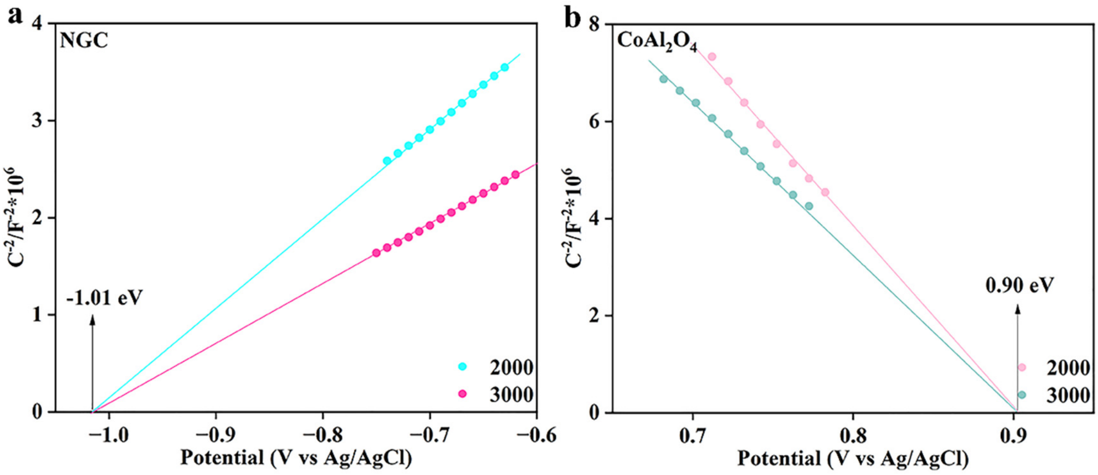

Figure S3. (a) Mott-Schottky curve of NGC; (b) Mott-Schottky curve of $CoAl_2O_4$.

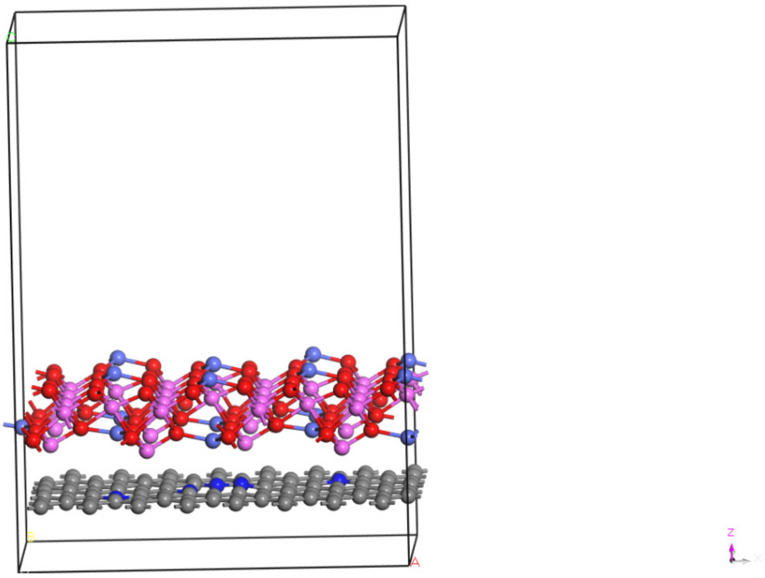

Fig.S4. Model for $CoAl_2O_4$@NGC

Table S2 Calculation formula of Gibbs free energy

Entry	Chemical Equations	Equations of adsorption energy
1	$CO_2 + e^- \rightarrow *CO_2^-$	$E_{ads} = E_{CoAl_2O_4@NGC + CO_2} - E_{CoAl_2O_4@NGC} - E_{CO_2}$
2	$*CO_2^- + H^+ + e^- \rightarrow *COOH$	$E_{ads} = E_{CoAl_2O_4@NGC + COOH} - E_{CoAl_2O_4@NGC + CO_2} - 1/2E_{H_2}$
3	$*COOH + H^+ + e^- \rightarrow *CO + H_2O$	$E_{ads} = E_{CoAl_2O_4@NGC + CO} + E_{H_2O} - E_{CoAl_2O_4@NGC + COOH} - 1/2E_{H_2}$
4	$*CO + H^+ + e^- \rightarrow *CHO$	$E_{ads} = E_{CoAl_2O_4@NGC + CHO} - E_{CoAl_2O_4@NGC + CO} - 1/2E_{H_2}$
5	$*CHO + H^+ + e^- \rightarrow *CHOH$	$E_{ads} = E_{CoAl_2O_4@NGC + CHOH} - E_{CoAl_2O_4@NGC + CHO} - 1/2E_{H_2}$
6	$*CHOH + H^+ + e^- \rightarrow *CH_2OH$	$E_{ads} = E_{CoAl_2O_4@NGC + CH_2OH} - E_{CoAl_2O_4@NGC + CHOH} - 1/2E_{H_2}$
7	$*CH_2OH + 3H^+ + 3e^- \rightarrow CH_4 + H_2O$	$E_{ads} = E_{CoAl_2O_4@NGC + CH_4} + E_{H_2O} - E_{CoAl_2O_4@NGC + CH_2OH} - 3/2E_{H_2}$
